# Dietary pattern characterized by a balanced diet rich in high-quality protein intake is associated with mild initial clinical manifestations in tuberculosis

**DOI:** 10.3389/fnut.2022.912703

**Published:** 2022-08-01

**Authors:** Xiaona Li, Zhaoyi Zhong, Yufeng Liu, Guifang Gong, Yangting Zhang, Yukang Wang, Chunchun Liu, Qiuzhen Wang

**Affiliations:** ^1^School of Public Health, Qingdao University, Qingdao, China; ^2^North Hospital of Qingdao Central Hospital, Qingdao, China; ^3^Qingdao Municipal Hospital, Qingdao, China; ^4^Chengyang District Center for Disease Control and Prevention, Qingdao, China

**Keywords:** dietary pattern, pulmonary tuberculosis, TB score, high-quality protein, concurrent TB and DM

## Abstract

**Background:**

The relationship between a single food or nutrient and pulmonary tuberculosis (TB) has been explored in many studies; however, the relationship between dietary patterns and TB is still lacking.

**Objective:**

Our study aims to investigate the association between dietary patterns and the initial clinical manifestations in patients with TB.

**Materials and methods:**

A cross-sectional study including 1,661 patients with active TB was conducted in Qingdao, China, from 2011 to 2019. A semiquantitative food frequency questionnaire was used to collect dietary data. Dietary patterns were determined by principal component factor analysis. Initial clinical manifestations were assessed using a combination of the patient self-reported clinical symptoms and the admission results indicated by the TB score. The associations between dietary patterns and TB scores in patients with TB were examined by the logistics regression model.

**Results:**

The analysis identified four dietary patterns: meat-fruit-seafood pattern; dairy-egg pattern; beans and their products-whole grain pattern; and refined grain-vegetable pattern. In a multiple-adjusted model, higher adherence to the meat-fruit-seafood pattern showed a protective effect on the TB score (OR 0.53, 95% CI 0.39, 0.84, *P* for trend = 0.010) and the association was stronger in patients older than 45 years (OR 0.32, 95% CI 0.16, 0.64, *P* for trend < 0.001). The higher adherence to beans and their products-whole grain pattern was a protective factor for TB score (OR 0.57, 95% CI 0.37, 0.87, *P* for trend = 0.025), and the association was also observed in patients with concurrent TB and diabetes mellitus (DM) with a more significant effect (OR 0.33, 95% CI 0.14, 0.80, *P* for trend = 0.025). No significant association was found between dairy-egg pattern and refined grain–vegetable dietary pattern with TB score.

**Conclusion:**

Dietary patterns characterized by a balanced diet rich in high-quality protein, sufficient energy, as well as marine n-3 PUFA, phytochemicals, B vitamins, and fiber are associated with mild initial clinical manifestations, and the association is stronger in patients older than 45 years and those with concurrent TB and DM.

## Introduction

Tuberculosis (TB), one of the ten leading causes of death worldwide, is caused by the bacillus *Mycobacterium tuberculosis* (*MTB*). Although the tuberculosis prevalence in the past few decades had been reduced in many countries, the burdens are still high, especially in low to middle-income countries. China has a high burden of TB. In 2020, China accounted for 8.5% of the global tuberculosis incidence, the second-highest in the world (1). Pulmonary tuberculosis prevention and treatment are still a vital public health issue.

Undernutrition is recognized as a driver of TB epidemics. Diet is an important factor affecting the nutritional status of patients with tuberculosis. A unique sociomedical experiment in the United Kingdom found adequate nutrition intervention nearly eliminated TB in children (2). During the Second World War, soldiers who were given an additional 1000 calories and 30 grams of protein in addition to the camp diet had a 93% reduction in tuberculosis incidence compared with soldiers who did not receive rations (3). A powerful, reverse, the exponential relationship between body mass index (BMI) and TB incidence has been shown in many large cohort studies (4–9). After adjusted for all confounders in 13211 adults, Cegielski et al. reported that new TB case incidence was 12 times higher in low BMI (BMI < 18.5) people compared with those with normal BMI (9).

Some studies have found that increased meat, vegetable, or fruit consumption is related to improving the cure rate of tuberculosis (10, 11). Although a variety of public health issues have been solved by the single-nutrient approach, many researchers believe it should turn to the dietary pattern approach for analysis because of the high relativity between individual food compositions (12, 13). Individual diet not only takes in a single food but also includes both nutritional and non-nutritional components. Therefore, examining food and nutrient intake indices that simultaneously indicate several relevant aspects of the diet may be more useful than focusing on single nutrient consumption (14).

Few studies use principal component analysis (PCA) to study the impact of dietary patterns on TB. PCA is a data-driven method, which reduces a large number of food variables to a small set to catch the main dietary characteristics of the population (15). PCA can be used to investigate the correlation of exposure to diseases in nutritional epidemiology. We utilized this method to analyze the dietary patterns and examine their links with the initial tuberculosis clinical manifestation. Our study aims to examine the link between dietary patterns and initial tuberculosis clinical manifestation. We hypothesize that a high-quality protein-rich diet is associated with mild initial clinical manifestations in patients with tuberculosis. Our study may provide evidence to support the recommendation of an optimal diet pattern for tuberculosis.

## Materials and methods

### Study population

From 2011 to 2019, 1,661 active patients with TB were randomly selected from a hospital in Qingdao. Among all the patients, 13 reported extreme energy intake (energy intake >5,000 kcal or <500 kcal) and 9 were too young (<16 years old). The final study sample consisted of 1,639 patients aged 16-88 years. The inclusion criteria were being (1) aged ≥16 years and (2) diagnosed as pulmonary tuberculosis by the clinician according to diagnostic criteria. The exclusion criteria were as follows: (1) HIV combined with cardiovascular, respiratory diseases, gastrointestinal, cancer, hepatopathy (such as hepatitis B or C, and alcoholic hepatitis), or mental diseases; (2) taking nutritional supplements 2 months before diagnosis; and (3) women in pregnancy or lactation stage.

The Ethics Committee of the Qingdao Center for Disease Control and Prevention approved the study. All participants provided informed consent.

### Data collection

To assure the authenticity and reliability of the interview, we conducted training for all interviewers before the formal investigation to master unified methods and skills, especially the attitude, investigation skills, and techniques. Trained interviewers collected detailed information including demographic and socioeconomic status (age, gender, habitation, education level, marital status, and income), living behaviors (smoking, alcohol consumption, and physical activity), medical history (such as hypertension and diabetes), and family medical history by using a structured questionnaire. According to the standardized procedure, the height and weight of each patient were measured on an empty stomach in the morning and wearing light clothes without shoes, and each item was repeated once. The weight scale used to measure the height and weight of patients was provided by Changzhou Wujin Weighing Instrument Co., Ltd. The minimum and maximum weights of the scale are, respectively, 5 kg and 200 kg. BMI was calculated using the formula BMI = weight (kg)/[height (m)]^2^ (16).

According to the diagnosis criteria for pulmonary tuberculosis (WS 288-2008, WS 288-2017), TB was diagnosed based on etiology combined with epidemiology, clinical manifestation, and chest radiography. Diabetes (DM) was diagnosed based on random blood glucose ≥11.1 mmol/L, fasting blood glucose ≥ 7.0 mmol/L, or self-reported diabetes.

The TB score was calculated according to the typical baseline signs and symptoms of tuberculosis, including cough, hemoptysis, tachycardia, dyspnea, chest pain, night sweating, anemia, expectoration, and fever (axillary temperature >37°C), and BMI. It was used to assess initial TB clinical severity (17–19). According to the tuberculosis scores of 0-2, 3-4, and >4, the patients were separated into three groups: mild, moderate, and severe. We sought the signs and symptoms of the patient’s case.

### Dietary assessment

During hospitalization, a semiquantitative food frequency questionnaire (FFQ) was used to evaluate dietary intake when patients were diagnosed with TB. The questionnaire is based on the food with a high frequency of intake, which was previously verified locally and developed by the China National Nutrition Survey (20, 21). All enrolled patients were required to answer the question about the dietary situation during the past 12 months. Food images (22) and models (23) were provided to patients to give the respondents a more intuitive concept of food quantification, to improve the accuracy of recall and investigation efficiency. These food images take different sizes of each food item and arrange them in ascending order on the coordinate paper marked with units of length. Next to the food, visual reference objects (such as a can of beer) familiar to people in daily life with fixed shapes and sizes are placed. In addition, the food model we adopted is a one-to-one imitation of food into a model that can be kept for a long time, and the weight of the food is indicated, eliminating the difference between picture vision and food vision. Food items were grouped *a priori* into 11 food categories (refined grains, whole grains, potatoes, beans, vegetables, fruits, red meat, poultry, dairy, eggs, and seafood) and dietary patterns were described using these food groups. The frequency of food consumption was divided into “times a day,” “times a week,” “times a month,” “times a year,” and “almost never”. The quantity of food intake was estimated by Chinese Liang (equivalent to 50 g).

### Statistical analysis

Dietary patterns were derived by PCA analysis with varimax rotation. To decrease the impact of higher frequency and greater variance food groups, each food group’s consumption frequencies were standardized by the mean and standard deviation before extraction of dietary patterns (24). Every dietary pattern was formed by using linear combinations of food groups. The coefficient defining these linear combinations, namely, factor loadings, represents the association between every food group and the corresponding pattern. Dietary patterns were described by food groups with loadings > 0.50. The factor score of every dietary pattern was calculated by adding the consumption frequency of every food group and weighing it according to the factor load of each patient. Further analysis used these scores for exposure variables. Factor analysis, a ‘*posteriori’* method, constructs all dietary patterns.

In this study, the characteristics of the participant were described by means ± SDs or percentages. Continuous variables were tested by analysis of variance, and categorical variables were tested using the chi-square test. After adjusting for age, BMI, diabetes, pulmonary cavity condition, initial treatment, and sputum smear examination, the association between the initial clinical manifestations and dietary pattern scores in patients with pulmonary tuberculosis was examined using a logistic regression model.

The obtained scores for each dietary pattern were divided into quartiles (quartile 1–quartile 4). In this study, we explored the association between dietary pattern and TB score and used individuals who had the lowest quartile of dietary pattern score during the past year as a reference. The median score of each dietary pattern was used to calculate *P* for trend, and the median was analyzed as a continuous variable in multivariate models. In addition, stratification by age (**≤**45 years or >45 years) and with or without concurrent diabetes was conducted to further examine whether dietary patterns have different effects on the characteristics of tuberculosis.

The confidence interval was 95%. In all statistical tests, *P* < 0.05 was used as 2-sided and significance. SPSS software version 20.0 was used for the statistical analyses.

## Results

### Dietary patterns

We extracted four main dietary patterns from factor analysis in this study. Table 1 shows the factor loadings of each dietary pattern. The first pattern represented the meat-fruit-seafood pattern, which explained 15.82% of the total variance of food intake. The characteristic of the first pattern was the frequent intake of red meat, poultry, fruits, and seafood. The second pattern represented the dairy-egg pattern, which explained 12.32% of the total variance. The characteristic of the second pattern was the frequent intake of dairy and eggs. The third pattern represented the beans and their products-whole grain pattern, which explained 11.59% of the total variance. The characteristic of the third pattern was the frequent intake of beans and their products, whole grains, poultry, and potatoes. The fourth pattern represented the refined grain-vegetable pattern, which explained 11.39% of the total variance. The characteristic of the fourth pattern was the frequent intake of refined grains and vegetables. The four patterns explained 51.11% of the total variation.

**TABLE 1:** Factor loadings for the four dietary patterns identified by PCA analysis in 1,639 patients with TB*.

	Dietary Pattern			
	Meat – fruit- seafood pattern	Dairy- egg pattern	Beans and their products -whole grain pattern	Refined grain – vegetable pattern
Refined grain	–	−0.115	0.114	0.822
Whole grain	–	–	0.663	–
Potato	0.302	−0.577	0.316	0.165
Vegetable	0.153	0.244	–	0.691
Fruit	0.593	0.296	−0.190	–
Red meat	0.686	−0.121	−0.143	0.137
Poultry	0.650	−0.199	0.232	–
Soy and their products	–	0.102	0.690	0.126
Dairy	–	0.680	0.160	–
Egg	0.177	0.563	0.261	0.137
Seafood	0.582	0.115	0.202	–
Variance explained (%)^#^	15.816	12.320	11.587	11.385

*Values are factor loadings.

Food groups are classified according to factor loadings. Absolute values of <0.10 not listed.

^#^ Percentage variance of total food intake explained by the patterns.

### Basic characteristics

Table 2 shows the distributions of the baseline characteristics of the patients by quartile of dietary pattern scores. Among all the participants, the mean age was 43.95 ± 16.87 years. Of the patients, 71% were men, 31.60% with an education level of undergraduate and above,46.30% reported smoking, and 32.80% reported alcohol drinking. Patients with a high meat-fruit-seafood pattern score are inclined to be younger, with a higher BMI and a higher educational level. Besides, higher refined grain-vegetable pattern scores tended to be younger age, more men, and higher educational levels.

**TABLE 2:** Distributions of tuberculosis characteristics by dietary pattern score quartiles 1,639 patients with tuberculosis*.

	All	Meat -fruit–		Dairy- egg		Beans and their products-		Refined grain –	
	participants	seafood pattern		pattern		whole grain pattern		vegetable pattern	
		Q1	Q4	Q1	Q4	Q1	Q4	Q1	Q4
**Baseline Characteristics**									
**Age, y**	43.95 ± 16.87	47.42 ± 17.32	43.38 ± 15.72	44.28 ± 17.42	45.53 ± 17.50	42.74 ± 16.59	44.71 ± 16.85	48.50 ± 17.06	42.13 ± 16.22
*P* †		<0.001		0.051		0.379		<0.001	
**Sex**									
Male (n,%)	1163, 71.00	291, 71.00	290, 70.70	321, 78.50	293, 71.60	282, 68.80	290, 70.90	272, 66.50	310, 75.80
Female (n,%)	476, 29.00	119, 29.00	120,29.30	88, 21.50	116, 28.40	128, 31.20	119, 29.10	137, 33.50	99, 24.20
*P* †		0.440		<0.001		0.013		0.012	
**BMI**, kg/m2‡	21.55 ± 3.29	21.37 ± 3.24	22.27 ± 3.56	21.70 ± 3.39	21.57 ± 3.41	21.46 ± 3.35	21.84 ± 3.19	21.62 ± 3.21	21.78 ± 3.69
*P* †		<0.001		0.717		0.211		0.285	
**Education** ‡									
Primary and lower (n,%)	263, 16.30	97, 24.10	67, 16.30	74,18.20	56, 13.70	55, 13.80	66, 16.20	91, 23.00	48, 11.70
Junior high school (n,%)	415, 25.70	105, 26.10	102, 24.90	100,24.60	112,27.50	110, 27.60	96, 23.60	107, 27.10	106, 25.90
Senior high school (n,%)	428, 26.50	92, 22.80	124, 30.20	112, 27.60	111, 27.20	114, 28.60	108, 26.50	90, 22.80	120, 29.30
Under-graduate and above (n,%)	510, 31.60	109, 27.00	117, 28.50	120, 29.60	129, 31.60	120, 30.10	137, 33.70	107, 27.10	135, 33.00
*P* †		<0.001		0.169		0.612		<0.001	
**Smokers** (n,%)‡	756, 46.30	197, 48.20	189, 46.20	200, 49.00	193, 47.20	190, 46.60	188, 46.00	191, 47.00	194,47.50
*P* †		0.788		0.446		0.584		0.868	
**Drinkers** (n,%)‡	533, 32.80	136, 33.40	133, 32.60	145, 35.80	134, 33.00	143, 35.00	139, 34.20	148, 36.50	125, 30.60
*P* †		0.843		0.344		0.384		0.291	

Q1, the lowest quartile; Q4, the highest quartile, *Dietary pattern scores obtained by multiplying factor loadings by corresponding standardized value of the frequency of intake of each food, and plus all these items. †ANOVA and χ^2^ tests were used to test the link of TB score and four quartiles of dietary pattern scores.

‡ Number of missing values for education level, drinkers, smokers, and BMI were 23, 12, 7, and 21, respectively.

### Relationship between dietary pattern and initial clinical manifestation

We summarized the multivariate regression analysis for the relationship between the quartile of dietary pattern scores and the initial clinical manifestations in patients with tuberculosis. We also present results stratified by age and whether or not patients had diabetes for all of the analyses we conducted, in addition to pooled results.

When comparing those in the highest quartile (quartile 4, Q4) with those in the lowest quartile (quartile 1, Q1) of the scores of respective dietary patterns, we observed the meat-fruit-seafood pattern was a protective factor of initial clinical manifestation in all patients with tuberculosis (OR 0.53, 95% CI 0.39, 0.84, *P* for trend = 0.010) after adjusted for the possible confounders. Similar associations were also observed in beans and their products-whole grain pattern (OR 0.57, 95% CI 0.37, 0.87, *P* for trend = 0.025) (refer to Table 3).

**TABLE 3:** Associations between dietary pattern score quartile and initial clinical manifestations.

	Q1	Q2	Q3	Q4	*P-trend*
**Moderate**					
**Meat-fruit–seafood pattern**					
OR (95%CI)	1.00 (ref)	1.05 (0.68, 1.64)	0.91 (0.58, 1.41)	0.79 (0.52, 1.20)	0.174
**Dairy-egg pattern**					
OR (95%CI)	1.00 (ref)	0.66 (0.43, 1.02)	0.55 (0.36, 0.85)	0.93 (0.60, 1.44)	0.782
**Beans and their products-whole grain pattern**					
OR (95%CI)	1.00 (ref)	0.58 (0.38, 0.90)	0.66 (0.43, 1.02)	0.57 (0.37, 0.87)	0.025
**Refined grain–vegetable pattern**					
OR (95%CI)	1.00 (ref)	0.79 (0.51, 1.23)	1.07 (0.68, 1.70)	1.05 (0.66, 1.64)	0.461
**Severe**					
**Meat-fruit–seafood pattern**					
OR (95%CI)	1.00 (ref)	0.65 (0.41, 1.05)	0.64 (0.40, 1.03)	0.53 (0.39, 0.84)	0.010
**Dairy-egg pattern**					
OR (95%CI)	1.00 (ref)	0.73 (0.45, 1.17)	0.82 (0.52, 1.29)	0.91 (0.57, 1.47)	0.850
**Beans and their products-whole grain pattern**					
OR (95%CI)	1.00 (ref)	0.82 (0.51, 1.32)	0.96 (0.60, 1.55)	0.72 (0.45, 1.15)	0.262
**Refined grain–vegetable pattern**					
OR (95%CI)	1.00 (ref)	0.66 (0.41, 1.06)	0.95 (0.58, 1.56)	0.88 (0.54, 1.43)	0.894

Q1–Q4, the lowest quartile to the highest quartile. ORs and 95% CIs were adjusted for age (continuous), BMI (discrete), pulmonary cavity condition (discrete), initial treatment (discrete), sputum smear examination (discrete), and diabetes(discrete).

Age-stratified analysis showed that after adjusted for the possible confounders, the highest quartile of the meat-fruit-seafood pattern was still a protective factor of initial TB clinical manifestation in patients aged >45 years with a more significant protective effect (OR 0.32, 95% CI 0.16, 0.64, *P* for trend <0.001). Also, in patients with concurrent TB and DM, beans and their products-whole grain pattern showed a more obvious association with initial TB clinical manifestation (OR 0.33, 95% CI: 0.14, 0.80, *P* for trend = 0.025) (Tables 4, 5).

**TABLE 4:** Associations between dietary pattern score quartile and initial clinical manifestations, stratified by age.

	Q1	Q2	Q3	Q4	*P-trend*
** ≤ 45 y**					
**Moderate**					
**Meat-fruit–seafood pattern**					
OR (95%CI)	1.00 (ref)	0.84 (0.46, 1.53)	0.95 (0.53, 1.70)	0.77 (0.43, 1.37)	0.436
**Dairy-egg pattern**					
OR (95%CI)	1.00 (ref)	0.56 (0.31, 0.99)	0.45 (0.25, 0.80)	0.65 (0.36, 1.17)	0.147
**Beans and their products-whole grain pattern**					
OR (95%CI)	1.00 (ref)	0.62 (0.35, 1.10)	0.78 (0.45, 1.36)	0.70 (0.40, 1.23)	0.319
**Refined grain–vegetable pattern**					
OR (95%CI)	1.00 (ref)	0.88 (0.46, 1.71)	1.31 (0.67, 2.54)	1.00 (0.52, 1.92)	0.793
**Severe**					
**Meat-fruit–seafood pattern**					
OR (95%CI)	1.00 (ref)	0.66 (0.34, 1.26)	0.83 (0.44, 1.56)	0.71 (0.38, 1.32)	0.431
**Dairy-egg pattern**					
OR (95%CI)	1.00 (ref)	0.65 (0.34, 1.23)	0.67 (0.36, 1.24)	0.72 (0.38, 1.38)	0.385
**Beans and their products-whole grain pattern**					
OR (95%CI)	1.00 (ref)	1.08 (0.58, 2.01)	1.29 (0.69, 2.40)	1.14 (0.61, 2.11)	0.616
**Refined grain–vegetable pattern**					
OR (95%CI)	1.00 (ref)	0.45 (0.23, 0.90)	0.80 (0.40, 1.59)	0.61 (0.31, 1.21)	0.555
** > 45 y**					
**Moderate**					
**Meat-fruit–seafood pattern**					
OR (95%CI)	1.00 (ref)	1.21 (0.62, 2.38)	0.70 (0.36, 1.39)	0.69 (0.37, 1.29)	0.107
**Dairy-egg pattern**					
OR (95%CI)	1.00 (ref)	0.86 (0.45, 1.66)	0.70 (0.37, 1.33)	1.41 (0.74, 2.71)	0.299
**Beans and their products-whole grain pattern**					
OR (95%CI)	1.00 (ref)	0.49 (0.24, 1.00)	0.53 (0.25, 1.09)	0.41 (0.21, 0.82)	0.024
**Refined grain–vegetable pattern**					
OR (95%CI)	1.00 (ref)	0.72 (0.39, 1.32)	0.85 (0.44, 1.64)	1.26 (0.64, 2.48)	0.398
**Severe**					
**Meat-fruit–seafood pattern**					
OR (95%CI)	1.00 (ref)	0.61 (0.30, 1.25)	0.38 (0.19, 0.79)	0.32 (0.16, 0.64)	<0.001
**Dairy-egg pattern**					
OR (95%CI)	1.00 (ref)	0.90 (0.44, 1.85)	1.04 (0.52, 2.08)	1.24 (0.61, 2.55)	0.501
**Beans and their products-whole grain pattern**					
OR (95%CI)	1.00 (ref)	0.55 (0.26, 1.20)	0.67 (0.31, 1.47)	0.42 (0.20, 0.89)	0.052
**Refined grain–vegetable pattern**					
OR (95%CI)	1.00 (ref)	0.83 (0.42, 1.61)	0.95 (0.47, 1.94)	1.24 (0.59, 2.59)	0.500

Q1–Q4, the lowest quartile to the highest quartile. ORs and 95% CIs were adjusted for BMI (discrete), pulmonary cavity condition (discrete), initial treatment (discrete), sputum smear examination (discrete), and diabetes(discrete).

**TABLE 5:** Associations between dietary pattern score quartile and initial clinical manifestations, stratified by concurrent diabetes.

	Q1	Q2	Q3	Q4	*P-trend*
**TB**					
**Moderate**					
**Meat-fruit–seafood pattern**					
OR (95%CI)	1.00 (ref)	1.12 (0.65, 1.94)	1.05 (0.61, 1.81)	0.78 (0.46, 1.31)	0.239
**Dairy-egg pattern**					
OR (95%CI)	1.00 (ref)	0.74 (0.44, 1.25)	0.57 (0.34, 0.95)	0.93 (0.54, 1.59)	0.598
**Beans and their products-whole grain pattern**					
OR (95%CI)	1.00 (ref)	0.74 (0.44, 1.24)	0.84 (0.50, 1.41)	0.64 (0.38, 1.07)	0.128
**Refined grain–vegetable pattern**					
OR (95%CI)	1.00 (ref)	0.71 (0.39, 1.31)	1.01 (0.55, 1.87)	0.87 (0.48, 1.60)	0.888
**Severe**					
**Meat-fruit–seafood pattern**					
OR (95%CI)	1.00 (ref)	0.81 (0.46, 1.44)	0.80 (0.45, 1.43)	0.54 (0.31, 0.94)	0.029
**Dairy-egg pattern**					
OR (95%CI)	1.00 (ref)	0.87 (0.49, 1.53)	0.82 (0.48, 1.42)	0.89 (0.50, 1.61)	0.664
**Beans and their products-whole grain pattern**					
OR (95%CI)	1.00 (ref)	1.17 (0.65, 2.09)	1.65 (0.93, 2.92)	1.10 (0.62, 1.95)	0.630
**Refined grain–vegetable pattern**					
OR (95%CI)	1.00 (ref)	0.64 (0.34, 1.21)	0.91 (0.48, 1.73)	0.67 (0.35, 1.28)	0.486
**TB-DM**					
**Moderate**					
**Meat-fruit–seafood pattern**					
OR (95%CI)	1.00 (ref)	0.88 (0.41, 1.92)	0.66 (0.30, 1.46)	0.77 (0.37, 1.59)	0.476
**Dairy-egg pattern**					
OR (95%CI)	1.00 (ref)	0.54 (0.25, 1.20)	0.53 (0.24, 1.15)	0.93 (0.44, 1.98)	0.762
**Beans and their products-whole grain pattern**					
OR (95%CI)	1.00 (ref)	0.33 (0.14, 0.76)	0.35 (0.15, 0.79)	0.38 (0.17, 0.85)	0.068
**Refined grain–vegetable pattern**					
OR (95%CI)	1.00 (ref)	0.86 (0.45, 1.66)	1.11 (0.54, 2.28)	1.47 (0.69, 3.13)	0.272
**Severe**					
**Meat-fruit–seafood pattern**					
OR (95%CI)	1.00 (ref)	0.38 (0.16, 0.90)	0.38 (0.16, 0.90)	0.47 (0.21, 1.05)	0.126
**Dairy-egg pattern**					
OR (95%CI)	1.00 (ref)	0.49 (0.20, 1.21)	0.80 (0.34, 1.91)	0.88 (0.38, 2.06)	0.793
**Beans and their products-whole grain pattern**					
OR (95%CI)	1.00 (ref)	0.40 (0.17, 0.99)	0.29 (0.12, 0.72)	0.33 (0.14, 0.80)	0.025
**Refined grain–vegetable pattern**					
OR (95%CI)	1.00 (ref)	0.60 (0.28, 1.26)	0.91 (0.41, 2.01)	1.40 (0.62, 3.20)	0.336

Q1–Q4, the lowest quartile to the highest quartile. ORs and 95% CIs were adjusted for age (continuous), BMI (discrete), pulmonary cavity condition (discrete), initial treatment (discrete), and sputum smear examination (discrete).

## Discussion

In this study, the meat-fruit-seafood pattern and beans and their products-whole grain pattern were found to be associated with mild initial clinical manifestations in patients with tuberculosis. The associations were discovered in patients with concurrent TB and DM, as well as those aged >45 years, with more pronounced effects.

The correlation between dietary factors and the risk of TB has been a field that has received great scientific interest. It has been reported that a vegetarian diet lacking meat and fish increases the risk of TB (25, 26), and protein intake lower than 50% of normal was closely related to a higher incidence of tuberculosis (27). Also, dietary marine n-3 PUFA was reported to be related to a dose-dependent reduction in the risk of active tuberculosis (28). However, the available reports were limited to vegetarian dietary patterns as well as certain nutrients. Wider dietary patterns and their correlations with TB need urgently to be investigated.

We found that the meat-fruit-seafood pattern was a protective factor of TB score, which is typical of high-quality protein, PUFA, polyphenols as well as a variety of minerals and vitamins such as Zn, niacin, vitamin B_12_, and Fe. In our opinion, the lower *MTB* burden, which is closely related to alleviated initial clinical manifestations, may be the main underlying mechanism. Protein deficiency may lead to a diminished number of macrophages, and impaired phagocytosis, and thus result in higher mycobacterial burdens (29, 30). Also, polyphenols, abundant in fruits, have been reported to have antituberculosis activity by inhibiting arabinogalactan synthesis in *MTB* cell walls (31–34). Another mechanism may be related to lowered inflammatory levels in the body. The study reported that docosahexaenoic acid has antibacterial and inflammation-resolving effects for tuberculosis (35). Consistently, *n*-3 PUFA can modulate the production of inflammatory cytokines in macrophages by inhibiting phosphorylation of I-κB in the NF-κB signaling pathway (36, 37). We found a more significant effect between the meat-fruit-seafood pattern and TB scores in patients older than 45 years. The risk of malnutrition increased with advancing age (38), especially for dietary protein, which is important for old people to sustain fair immunity. Aging is also related to a significant rise in oxidative damage and serum levels of inflammatory markers (39), and vitamins (such as vitamin C and E) and phytochemicals in fruit were highlighted as important for reducing oxidative stress in elderly patients (40). These can partially explain the higher odds ratio observed in patients older than 45.

We also found beans and their products-whole grain pattern were a protective factor of initial TB clinical manifestation. This diet also provides patients with high-quality protein, as we discussed above. Also, it is rich in flavonoids, B vitamins, and dietary fiber. Flavonoids, rich in beans, can play an antituberculosis role by inhibiting the *Mycobacteria* proteasome (41). Whole grains are an important source of B vitamins, including thiamin, riboflavin, niacin, and so on. A study reported that thiamin promoted the immune response by limiting the survival of *Mycobacterium tuberculosis* in macrophages and *in vivo* by regulating peroxisome proliferator-activated receptor γ (42). Therefore, lowered initial *MTB* burden may also be the key underlying mechanism. Also, the composition of essential amino acids of cereal protein is not reasonable, and the mixture of beans can play a complementary role in protein. In addition, the protective effect of this dietary pattern was more significant in patients with concurrent TB and DM. Beans and whole grains are beneficial to people with diabetes because of high fiber and low glycemic index, making diabetics assist in maintaining healthy blood glucose and insulin levels (43–45). Consistently, we found a more significant association in patients with concurrent diabetes.

Dairy-egg patterns and refined grain–vegetable patterns were not observed to be related to TB scores in this study. The result of the dairy-egg pattern was somewhat different from what we hypothesized. However, although dairy and eggs are sources of high-quality protein, this diet is not optimal for tuberculosis, mainly due to insufficient energy intake. As we know, protein-energy malnutrition is prevalent in patients with tuberculosis, which is closely correlated with the clinical manifestation of the patient (46). Therefore, a balanced diet with sufficient energy and protein may be important to patients with TB. A refined grain–vegetable pattern does not provide sufficient high-quality protein. In addition, the nutritional value of refined grain was decreased due to endosperm deficiency in the process of processing, leading to the loss of B vitamins as well as some essential fatty acids.

As far as we know, this is the first study to explore the association between normal dietary patterns and the initial clinical manifestations in patients with tuberculosis. We provide evidence of nutrition and TB based on dietary patterns, which is easier to translate into public health practice as well as food policy. In addition, 33.5% of patients in this study had concurrent diabetes. This allows us to further analyze the correlation between dietary patterns and TB scores in this subgroup, which is of great significance due to the increasing double burden of TB and DM in low to middle-income countries, including China. However, some limitations need to be recognized in this study. First, dietary intakes were self-reported, which inevitably leads to false positives due to recall bias. However, food models and images were provided to patients to reduce recall bias. Second, under different conditions of cooking and processing of the food, the absorption, and bioavailability of the nutrients in the body may differ in patients with TB. Third, the research was conducted in Qingdao, a coastal city; hence, the dietary habits of patients with TB may differ to a certain extent from those living in inland cities. However, our results may provide more precise dietary guidelines for coastal dwellers.

## Conclusion

In conclusion, the consumption of a dietary pattern characterized by a balanced diet rich in high-quality protein, sufficient energy, as well as marine n-3 PUFA, phytochemicals, B vitamins, and fiber is associated with mild initial clinical manifestations in patients with tuberculosis, especially in patients with older age and concurrent DM. Our findings provide important evidence on an optimal diet pattern for patients with tuberculosis. Further large-scale studies on patients with TB, especially in inland populations, are needed.

## Data availability statement

The original contributions presented in this study are included in the article/supplementary material, further inquiries can be directed to the corresponding author/s.

## Ethics statement

The studies involving human participants were reviewed and approved by The Ethics Committee of Qingdao Disease Prevention and Control Centre. Written informed consent to participate in this study was provided by the participants’ legal guardian/next of kin.

## Author contributions

QW and YL conceptualized the topic. YL assisted in organizing field work and participated in the investigation. YW and CL conducted the dietary survey and participated in the site work. ZZ, YZ, and GG arranged the questionnaire and entered the data. XL statistical analysis of relevant data and wrote the original draft. QW helped to review the manuscript. All authors have read and approved the final manuscript.
